# Prevalence of diarrhoea and treatment-seeking practices among children <2 years of age in the Birhan cohort, Ethiopia, 2018–19

**DOI:** 10.7189/jogh.14.04181

**Published:** 2024-11-01

**Authors:** Gedefaw Abeje Fekadu, Damen Hailemariam, Muluemebet Abera, Firmaye Bogale Woldie, Bezawit Mesfin Hunegnaw, Clara Pons-Duran, Robera Olana Fite, Kassahun Alemu, Lisanu Taddesse, Delayehu Bekele, Getachew Tolera, Grace J Chan

**Affiliations:** 1 Health System and Reproductive Health Research Directorate, Ethiopian Public Health Institute, Addis Ababa, Ethiopia; 2 Department of Reproductive Health and Population Studies, School of Public Health, College of Medicine and Health Sciences, Bahir Dar University, Bahir Dar, Ethiopia; 3 School of Public Health, College of Health Sciences, Addis Ababa University, Addis Ababa, Ethiopia; 4 Department of Population and Family Health, Faculty of Public Health, Institute of Health, Jimma University, Jimma, Ethiopia; 5 Knowledge Translation Directorate, Ethiopian Public Health Institute, Addis Ababa, Ethiopia; 6 Department of Paediatrics and Child Health, Saint Paul’s Hospital Millennium Medical College, Addis Ababa, Ethiopia; 7 Department of Epidemiology, Harvard T.H. Chan School of Public Health, Boston, Massachusetts, USA; 8 HaSET Maternal and Child Health Research Program, Addis Ababa, Ethiopia; 9 Department of Gynaecology and Obstetrics, Saint Paul’s Hospital Millennium Medical College, Addis Ababa, Ethiopia; 10 Research and Technology Transfer Directorate, Ethiopian Public Health Institute, Addis Ababa, Ethiopia; 11 Department of Paediatrics, Boston Children's Hospital, Harvard Medical School, Boston, USA

## Abstract

**Background:**

Estimating the proportion of children with diarrhoea and those who are taken in as inpatients or outpatients is important for policy planning, resource allocation, and to evaluate the effectiveness of diarrhoea prevention and control interventions. We aimed to estimate the proportion of children <2 years of age with diarrhoea, explore their treatment-seeking practices, and identify factors associated with both diarrhoea and treatment seeking.

**Methods:**

We designed a longitudinal study based on a sample of children <2 years of age in the Birhan field site from September 2018 to September 2019. The study site collected data on child mortality and morbidity and treatment-seeking practice for those with a history of illness every three months. Mothers/caregivers were asked about signs or symptoms of illnesses for a two-week period prior to each study visit. We estimated the proportion of children <2 years of age with diarrhoea and treatment-seeking practices for each of the four rounds of data collection and identified associated factors through bivariable and multivariable logistic regression.

**Results:**

We enrolled 4678 children <2 years of age. The proportion of children with diarrhoea was the highest from 11 September 2018 to 9 December 2018 (4.47%; 95% confidence interval (CI) = 3.70–5.35) and the lowest from 10 December 2018 to 9 March 2019 (2.48%; 95% CI = 1.90–3.19). Children from households with chlorinated drinking water had a 50% (adjusted odds ratio (aOR) = 0.50; 95% CI = 0.28–0.88) lower odds of developing diarrhoea compared to those who did not. Among 339 children with diarrhoea, 275 (81.12%; 95% CI = 76.54–85.15) were taken to health facilities for treatment. Female children had lower odds of being taken to health facilities for treatment (aOR = 0.37; 95% CI = 0.17–0.80) compared to males.

**Conclusions:**

While the proportion of children with diarrhoea in our study was lower than that observed in prior research conducted in Ethiopia, treatment-seeking practices were higher. Female children and children from the poorest families had lower odds of treatment. We recommend more studies to explore gender-based and socioeconomic differences affecting treatment-seeking practices.

The World Health Organization (WHO) defines diarrhoea as the passage of three or more loose or liquid stools per day [1]. It is usually a symptom of an infection in the intestinal tract, which could be bacterial, viral, or parasitic [1,2]. Diarrhoea may cause severe dehydration and malnutrition which increases the risk of death [1,3]. According to various studies conducted in Ethiopia between 2016 and 2019, its prevalence among children <5 years varied from 11% to 23% [4]. In 2019, diarrhoea was the second leading cause of mortality and morbidity for Ethiopian children [5,6] and the leading cause of death among children ≥12 months [7,8].

A pregnancy outcome study from 2015–20 in Eastern Ethiopia indicated that diarrhoea was the first among the top ten causes of <5 mortality [9]. A systematic review in Ethiopia identified breastfeeding, hand washing practices, availability of toilets, maternal educational status, residence, distance from drinking water source, and age of the child as factors associated with diarrhoea disease among children [10]. Studies based on the 2016 Ethiopian Demographic and Health Survey (DHS) data identified child’s age, birth order, being twin, birth weight, breastfeeding, residence, source of drinking water, antenatal care attendance and availability of latrine as predictors of diarrhoea among children [11,12].

Focussing on known risk factors and promoting access to and use of oral rehydration solution (ORS) could reduce diarrhoea-associated morbidity and mortality [1]. However, diarrhoea-related deaths largely result from delayed or absence of medical treatment [13]. Per the 2016 Ethiopian DHS data, maternal age, residence, sex of the child, exposure to mass media, information about oral rehydration, fertility desire, wealth index, and region were found to be associated with treatment-seeking practices for children with diarrhoea [14].

Most previous studies conducted in Ethiopia included children <5 years of age. However, as the early childhood period determines the quality of health and well-being in later life, it is crucial to study the proportion of children with diarrhoea and treatment-seeking practice for children as early as possible. Estimating the burden of diarrhoeal disease in children <2 years of age and determining their treatment-seeking practices could be useful in evaluating the effectiveness of diarrhoea prevention and control interventions, and could help reduce diarrhoea-related mortality and morbidity among this sensitive population. We hence conducted this analysis to determine the proportion of children <2 years of age with diarrhoea and their treatment-seeking practice for diarrhoea, as well as to identify any associated factors.

## METHODS

### Study design and setting

We conducted a secondary analysis of data collected from the Birhan field site from 11 September 2018 to 10 September 2019. The site is located in the Amhara region of Ethiopia, approximately 130 km from Addis Ababa, and was established as a sentinel field site to generate evidence on maternal and child health. It consists of two districts, Angolela Tera and Kewet, in Amhara Region, covering a total area of 636 km^2^. Each district has eight *kebeles* (the lowest administration unit); of the total 16 *kebeles*, two are urban and 14 are rural [15].

### Data collection process and study population

The Birhan study team conducted a baseline census in 2018 to collect demographic and health data of women of reproductive age and children <2 years of age. Data collectors visited each household and asked mothers/caretakers to recall clinical symptoms of diarrhoea (its duration, the presence of blood in the stool), and the type and duration of treatment provided (if given) within the past two weeks [15]. Similar data were collected every three months from 2018 to 2019 in four rounds using an open data kit (ODK), which was developed and customised for longitudinal and relational data collection.

We included all children <2 years of age from the four rounds to estimate the proportion of children with diarrhoea and determine their treatment-seeking practices, as well as to identify any factors associated with either outcome.

### Operational definitions

We defined diarrhoea as the passage of three or more loose or liquid stools per day (or more frequent passage than is normal for the individual) in the last two weeks, as per the WHO definition [1]. For example, the child was considered to have diarrhoea if the mother/caregiver reported a history of diarrhoea during one of the two-week recall periods. Treatment-seeking practices were based on whether the child was taken to health facilities for treatment of diarrhoea at least during one of the reported episodes. Occurrence of diarrhoea and treatment-seeking practices were measured based on the mothers’/caregivers’ responses during study visits.

### Variable and measurements

The primary outcome variables for this study were diarrhoea among children <2 years of age and treatment-seeking practice for children with diarrhoea. The exposure variables included were household and child characteristics (age, parents’ religion, age, sex, wealth index and residence, source of income, and mobile ownership), housing characteristics (ownership of the house and number of household members) household water, hygiene, and sanitation conditions (presence of waste piles near the house, source and location of drinking water, drinking water treatment, type of toilet facility, access to soap for hand washing, and method of waste disposal).

### Data analysis

We determined the overall period prevalence of children with diarrhoea by dividing the sum of children who had a history of diarrhoea in at least one of the four data collection rounds. We then calculated the proportion of children with diarrhoea for each round using the same approach. Similarly, treatment-seeking practices for diarrhoea were determined for affected children for each round by dividing the number of children that were taken to a health facility by all children with a history of diarrhoea. We computed descriptive statistics, including frequencies and cross tabulations, to describe the study population and to present the caregivers’ sociodemographic, housing, and environmental characteristics. Lastly, we used binary and multivariable logistic regression to identify factors associated with diarrhoea and related treatment-seeking practice in the study population. For the bivariable regression model, we computed correlation coefficients to rule out multicollinearity among exposure variables.

All analyses were done in Stata, version 17 (StataCorp LLC, College Station, TX, USA).

## RESULTS

### Characteristics of children

We included 4678 children in the analysis: 2650 in round one, 2428 in round two, 2015 in round three, and 2336 in round four, with respective mean ages of 12.44, 13.11, 8.40, and 10.24 months. Most children (97.35% in round one and 92.46% in round 4) received the first dose of the Rota vaccine (**Table 1**).

**Table 1:** Age, vaccination history, and history of illness by survey time among children <2 y of age, Birhan HDSS 2018–19*

	Time of data collection, n (%)			
**Characteristics**	**11 September 2018 to 9 December 2018**	**10 December 2018 to 9 March 2019**	**10 March 2019 to 7 June 2019**	**8 June 2019 to 10 September 2019**
Age in months				
*<6*	566 (22.59)	313 (13.36)	688 (34.37)	607 (26.41)
*6–11*	605(24.14)	788 (33.65)	797(39.81)	740 (32.20)
*12–24*	1335 (53.27)	1241 (52.99)	517 (25.82)	951 (41.38)
*Range*	0.00–23.96	0.00–23.96	0.00–23.96	0.33–23.6
*x̄*	12.44	13.11	8.4	10.24
*Total*	2506	2342	2002	2311
Has ever been vaccinated				
*No*	311 (12.24)			205 (8.91)
*Yes*	2229 (87.76)			2097 (91.09)
*Total*	2540			2302
History of illness				
*Children*	312 (11.77)	207 (8.53)	214 (10.62)	185 (7.92)
*Total*	2650	2428	2015	2336
Received first dose of Rota vaccine				
*No*	25 (2.65)			58 (7.54)
*Yes*	919 (97.35)			711 (92.46)
Total	944			769
Received second dose of Rota vaccine				
*No*	102 (10.81)			122 (15.86)
*Yes*	842 (89.19)			647 (84.14)
*Total*	944			769
History of diarrhoea				
*Children*	113 (4.47)	59 (2.48)	76 (3.81)	78 (3.39)
*Total*	2527	2379	1994	2300
Blood in diarrhoea				
*Yes*	22 (19.47)	5 (8.47)	4 (5.26)	9 (11.54)
*No*	91 (80.53)	54 (91.53)	72 (94.74)	69 (88.46)
*Total*	113	59	76	78

x̄ – mean

*Presented as n (%) unless specified otherwise.

In total, 48.10% (n = 2250) children were female, 80.29% (n = 3755) were rural residents, and 90.68% (n = 3027) were from the Amhara ethnic group. Of all the children whose parents responded to sources of drinking water, 66.07% (n/N = 3084/4668) had improved water sources and 36.86% (n/N = 1707/4668) had improved toilet facilities. Approximately 36.26% (n/N = 1696/4677) of children were from households in the lowest wealth tertile, while families with their own income comprised more than 97% (n/N = 4547/4657) of all households. Farming was the main source of income for 79.45% (n/N = 2636/3318) households. Approximately 85% (n/N = 3992/4668) of children were living in households in which the parents owned their house, while the mean family size where these children were living was 4.79 (standard deviation (SD) = 1.85). At least one member of a household owned a mobile phone in 76.34% (n/N = 3555/4657) of households; 49% (n/N = 2285/4657) of households had at least one member who owned a bank account. The mean estimated walking time to the nearest health facility was 65.74 (SD = 54.05) minutes. Regarding vaccination, approximately 97% (n/N = 40519/4190) of the children were vaccinated at least once, with 95.79% (n/N = 1935/2020) receiving the first and 91.79% (n/N = 1844/2009) receiving the second dose of the Rota vaccine (Table S1 in the **Online Supplementary Document**).

### Period prevalence of diarrhoea

In the two weeks before the date of the survey, 18.96% (n/N = 887/4668) children had a history of illness, with 81.40% (n/N = 722/887) reporting one episode of illness. When asked about diarrhoea, 7.25% (n/N = 339/4678; 95% CI = 6.52–8.01) reported having a history of diarrhoea. From these, 89.38% (n = 303) of parents reported one episode of diarrhoea, while 12.93% (n = 42) reported having blood in the stool (**Table 2**).

**Table 2:** History of illness and diarrhoea among children <2 years of age, Birhan HDSS, Ethiopia, 2018–19*

Variables	n (%)
Residence (n = 4677)	
*Urban*	922 (19.71)
*Rural*	3755 (80.29)
Agro-ecology (n = 4677)	
*Highland*	1791 (38.29)
*Lowland*	2889 (61.71)
Ethnicity (n = 3338)	
*Amhara*	3027 (90.68)
*Oromo*	202 (6.05)
*Other*	109 (3.27)
Wealth index (n = 4677)	
*First tertile (least wealthy)*	1696 (36.26)
*Second tertile*	1636 (34.98)
*Third tertile (wealthiest)*	1345 (28.76)
Household member with bank account (n = 4657)	
*No*	2358 (50.63)
*Yes*	2285 (49.07)
*Unknown*	14 (0.3)
Family has own income (n = 4657)	
*No*	110 (2.36)
*Yes*	4547 (97.64)
Main source of income or livelihood (n = 3318)	
*Government employment*	141 (4.25)
*Private employment*	52 (1.57)
*NGO employment*	30 (0.9)
*Farming*	2636 (79.45)
*Petty trader*	62 (1.87)
*Merchant*	105 (3.16)
*Daily labor*	218 (6.57)
*Other*	74 (2.23)
Ownership of the house (n = 4668)	
*Own*	3992 (85.58)
*Rented from government*	50 (1.07)
*Rented from individual*	590 (12.64)
*Other*	36 (0.77 =
Waste pile near the home (n = 4668)	
*No*	4191 (89.78)
*Yes*	477 (10.22)
Family member owned mobile phone (n = 4657)	
*No*	1102 (23.66)
*Yes*	3555 (76.34)
Source of drinking water (n = 4668)	
*Within premises*	726 (15.55)
*Improved*	3084 (66.07)
*Unimproved*	524 (11.23)
*Surface water*	334 (7.16)
Location of drinking water source (n = 4668)	
*Own dwelling*	26 (0.56)
*Own compound*	762 (16.32)
*Outside the compound*	3880 (83.12)
Drinking water treated (n = 4668)	
*Yes*	1599 (34.25)
*No*	3069 (65.75)
Drinking water treated by boiling (n = 1599)	
*No*	1531 (96.12)
*Yes*	62 (3.88)
Drinking water treated by chlorine (n = 1599)	
*No*	557 (34.83)
*Yes*	1042 (65.17)
Drinking water treated by iodine (n = 1599)	
*No*	1509 (94.37)
*Yes*	90 (5.63)
Drinking water treated by filtration (n = 1599)	
*No*	1222 (76.42)
*Yes*	377 (23.58)
Water treatment options (n = 1599)	
*None of the above*	185 (11.57)
*At least one of the above*	1414 (88.43)
Type of toilet facility (n = 4668)	
*Improved*	1707 (36.57)
*Unimproved*	1730 (37.06)
*Open*	1231 (26.37)
Access to soap (n = 4668)	
*No*	540 (11.57)
*Yes*	4128 (88.43)
Refuse disposal (n = 4668)	
*Pit*	879 (18.83)
*Open field*	1170 (25.06)
*Burn*	1226 (26.26)
*Compost*	672 (14.4)
*Municipality collection*	86 (1.84)
*Farm*	619 (13.26)
*Other*	16 (0.34)
Estimated walking time in minutes to the nearest health facility (n = 4668), x̄ (SD)	65.74 (54.05)
Family size (n = 3941), x̄ (SD)	4.79 (1.85)
Vaccinated at least once (n = 4190)	
*No*	139 (3.32)
*Yes*	4051 (96.68)
Vaccinated with first dose of Rota vaccine (n = 2020)	
*No*	85 (4.21)
*Yes*	1935 (95.79)
Vaccinated with second dose of Rota vaccine (n = 2009)	
*No*	165 (8.21)
*Yes*	1844 (91.79)

SD – standard deviation, x̄ – mean

*Presented as n (%) unless specified otherwise.

The proportion of children with diarrhoea varied over time and showed a significant decline, being the highest (4.47%; 95% CI = 3.70–5.35) in a survey conducted from 11 September 2018 to 9 December 2018 and the lowest (2.48%; 95% CI = 1.90–3.19) in round two, which was conducted from 10 December 2018 to 9 March 2019. Over half (57.23%) of the children with diarrhoea were male, with 85.55% being from rural areas. Almost half (46.90%) of the children with diarrhoea were from households in the second wealth tertile (Table S2 in the **Online Supplementary Document**). There was a statistically significant difference among children with diarrhoea by sex of the child (*P* < 0.05), residence (*P* < 0.05), source of income (*P* < 0.05), owning of mobile phone (*P* < 0.01), treatment of drinking water (*P* < 0.01), and vaccination status regarding second dose of Rota vaccine (*P* < 0.05) (**Table 3**).

**Table 3:** Association of housing, child, and caregiver characteristics with diarrhoea among children <2 years of age, Birhan HDSS, 2018–19

	History of diarrhoea in at least one of the rounds		
**Characteristics**	**No, n (%)**	**Yes, n (%)**	*P*-value*
Sex (n = 4678)			0.042
*Male*	2234 (92.01)	194 (7.99)	
*Female*	2105 (93.56)	145 (6.44)	
Residence (n = 4677)			0.011
*Urban*	873 (94.69)	49 (5.31)	
*Rural*	3465 (92.28)	290 (7.72)	
Ethnicity (n = 3338)			0.20
*Amhara*	2796 (92.37)	231 (7.63)	
*Oromo*	192 (95.05)	10 (4.95)	
*Other*	104 (95.41)	5 (4.59)	
Wealth index (n = 4677)			<0.01
*First tertile (least wealthy)*	1599 (94.28)	97 (5.72)	
*Second tertile*	1477 (90.28)	159 (9.72)	
*Third tertile (wealthiest)*	1262 (93.83)	83 (6.17)	
A member of the household has bank account (n = 4657)			0.13
*No*	2202 (93.38)	156 (6.62)	
*Yes*	2104 (92.08)	181 (7.92)	
*Unknown*	14 (100.00)	0 (0.00)	
Family has own income (n = 4657)			0.72
*No*	103 (93.64)	7 (6.36)	
*Yes*	4217 (92.74)	330 (7.26)	
Main source of income or livelihood (n = 3318)			0.03
*Non-farming*	645 (94.57)	37 (5.43)	
*Farming*	2429 (92.15)	207 (7.85)	
Ownership of the house (n = 4668)			0.54
*Not owned*	631 (93.34)	45 (6.66)	
*Owned*	3700 (92.69)	292 (7.31)	
Waste pile near the home (n = 4668)			0.07
*No*	3898 (93.01)	293 (6.99)	
*Yes*	433 (90.78)	44 (9.22)	
A member of the household own mobile phone (n = 4657)			<0.01
*No*	1044 (94.74)	58 (5.26)	
*Yes*	3276 (92.15)	279 (7.85)	
Source of drinking water (n = 4668)			<0.01
*Pipe within premises*	695 (95.73)	31 (4.27)	
*Improved*	2819 (91.41)	265 (8.59)	
*Unimproved*	502 (95.8)	22 (4.20)	
*Surface water*	315 (94.31)	19 (5.69)	
Location of drinking water source (n = 4668)			<0.01
*Own dwelling*	23 (88.46)	3 (11.54)	
*Own compound*	731 (95.93)	31 (4.07)	
*Outside the compound*	3577 (92.19)	303 (7.81)	
Treat drinking water (n = 4668)			<0.01
*No*	2875 (93.68)	194 (6.32)	
*Yes*	1456 (91.06)	143 (8.94)	
Treat drinking water by boiling (n = 1599)			0.25
*No*	1397 (90.89)	140 (9.11)	
*Yes*	59 (95.16)	3 (8.84)	
Use chlorine to treat drinking water (n = 1599)			0.83
*No*	506 (90.84)	51 (9.16)	
*Yes*	950 (91.17)	92 (8.83)	
Use iodine to treat drinking water (n = 1599)			0.12
*No*	1370 (90.79)	139 (9.21)	
*Yes*	86 (95.56)	4 (4.44)	
Filter to treat your drinking water (n = 1599)			0.24
*No*	1107 (90.59)	115 (9.41)	
*Yes*	349 (92.57)	28 (7.43)	
Used at least one water treatment options (n = 1599)			0.01
*No*	159 (85.95)	26 (14.05)	
*Yes*	1297 (91.73)	117 (8.27)	
Type of toilet facility (n = 4668)			0.52
*Improved*	1582 (92.68)	125 (7.32)	
*Unimproved*	1614 (93.29)	116 (6.71)	
*Open*	1135 (92.20)	96 (7.80)	
Access to soap (n = 4668)			0.22
*No*	508 (94.07)	32 (5.93)	
*Yes*	3823 (92.61)	305 (7.93)	
Refuse disposal (n = 4668)			0.83
*Unsafe*	2814 (92.84)	217 (7.16)	
*Safe*	1517 (92.67)	120 (7.33)	
Vaccinated at least once (n = 4190)			0.22
*No*	132 (94.96)	7 (5.04)	
*Yes*	3732 (92.13)	319 (7.87)	
First dose of Rota vaccine (n = 2020)			0.29
*No*	81 (95.29)	4 (4.71)	
*Yes*	1784 (92.20)	151 (7.80)	
Second dose of Rota vaccine (n = 2009)			0.05
*No*	159 (96.36)	6 (3.64)	
*Yes*	1698 (92.08)	146 (7.92)	

*χ^2^ test.

### Treatment-seeking practices for diarrhoea

Of the 339 children who had a history of diarrhoea, treatment was sought for 275 (81.12%; 95% CI = 76.54–85.15), with all caregivers reporting that treatment was sought from health facilities. The proportion of children taken to health facilities for diarrhoea treatment ranged from 76.92% (data collected during round four (8 June to 10 September 2019)) to 83.05% (data collected during round two (10 December 2018 to 9 March 2019)). However, the difference was not statistically significant (**Table 1**). In the between-group analysis, only the wealth index was statistically significant for treatment-seeking practice for diarrhoea (**Table 4**). Relatively few children with diarrhoea were given ORS. There was no difference in the amount of food and fluids given during the illness by round of data collection (**Table 5**).

**Table 4:** Selected housing, child, and caregiver characteristics by treatment for diarrhoea among children <2 years of age, Birhan field site, 2018–19

	Treated for diarrhoea		
**Variable**	**No, n (%)**	**Yes, n (%)**	***P*-value**
Sex (n = 339)			0.19
*Male*	32 (16.49)	162 (83.51)	
*Female*	32 (22.07)	113 (77.93)	
Residence (n = 339)			0.37
*Urban*	7 (14.29)	42 (85.71)	
*Rural*	57 (19.66)	233 (80.34)	
Ethnicity (n = 246)			0.98
*Amhara*	41 (17.75)	190 (82.25)	
*Oromo*	2 (20.00)	8 (80.00)	
*Other*	1 (20.00)	4 (80.00)	
Wealth index (n = 339)			<0.01
*First tertile (the least)*	31 (31.96)	66 (68.04)	
*Second tertile*	27 (16.98)	132 (83.02)	
*Third tertile (the wealthiest)*	6 (7.23)	77 (92.77)	
One member of the household has bank account (n = 377)			0.08
*No*	36 (23.08)	120 (76.92)	
*Yes*	28 (15.47)	153 (84.53)	
Family has own income (n = 377)			0.51
*No*	2 (28.57)	5 (71.43)	
*Yes*	62 (18.79)	268 (81.21)	
Ownership of the house (n = 377)			
*Not own*	6 (13.33)	39 (86.67)	
*Own*	58 (19.68)	234 (80.14)	
Owning mobile phone by member of the household (n = 377)			
*No*	12 (20.69)	46 (79.31)	
*Yes*	52 (18.64)	227 (81.36)	
Main source of income (n = 244)			0.21
*Non-farming*	4 (10.81)	33 (89.19)	
*Farming*	40 (18.03)	167 (81.97)	
Blood in diarrhoea (n = 339)			0.145
*No*	60 (20.34)	235 (79.66)	
*Yes*	4 (9.09)	40 (90.91)	

*χ^2^ test.

**Table 5:** Proportion of children <2 years of age treated for diarrhoea and those given ORS, liquids, or food during the diarrhoea episode, Birhan field site, 2018–19

Time of survey	Proportion of children treated for diarrhoea, % (95% CI)	Proportion of children given ORS, % (95% CI)	Proportion of children given the same or higher amounts of liquids than usual, % (95% CI)	Proportion of children given the same or higher amounts of food than usual, % (95% CI)
11 September to 9 December 2018	78.76 (70.07–85.89)	18.58 (11.89–26.99)	34.82 (26.07–44.40)	26.61 (18.60–35.93)
10 December 2018 to 9 March 2019	83.05 (71.03–91.56)	7.02 (1.95–17.00)	44.07 (31.16–57.60)	37.29 (25.04–50.85)
10 March to 7 June 2019	80.26 (69.54–88.51)	14.67 (7.56–24.73)	46.05 (34.55–57.87)	36.84 (26.06–48.69)
8 June to 10 September 2019	76.92 (66.00–85.71)	12.99 (6.41–22.59)	27.27 (17.74–38.62)	20.78 (12.37–31.54)

CI – confidence interval

### Factors associated with diarrhoea

In the bivariable analysis, the sex of the child (crude odds ratio (cOR) = 1.26; 95% CI = 1.01–1.58), wealth index, being in the second tertile (cOR = 1.77; 95% CI = 1.36–2.31), source of income (cOR = 1.48; 95% CI = 1.04–2.13), ownership of mobile phone by household member (cOR = 1.21; 95% CI = 0.97–1.52), and source of drinking water (cOR = 2.11; 95% CI = 1.44–3.09) were significantly associated with diarrhoea among children <2. In the multivariable logistic regression analysis, we found that children whose parents use chlorine to treat drinking water had 50% lower odds of having diarrhoea compared to children whose parents did not use chlorine (adjusted odds ratio (aOR) = 0.50; 95% CI = 0.28–0.88), while children who were from households who had access to hand washing soap had 8.49 (aOR = 8.49; 95% CI = 1.15–62.73) higher odds of developing diarrhoea compared to children from households with no access to soap for hand washing (**Table 6**).

**Table 6:** Factors associated with diarrhoea among children <2 years of age in Birhan field site, 2018–19

	History of diarrhoea in at least one of the rounds			
**Variables**	**No, n (%)**	**Yes, n (%)**	**cOR (95% CI)**	**aOR (95% CI)**
Sex (n = 4678)				
*Male*	2234 (92.01)	194 (7.99)	1.26 (1.01–1.58)	1.59 (0.99–2.54)
*Female*	2105 (93.56)	145 (6.44)	1	1
Residence (n = 4677)				
Urban	873 (94.69)	49 (5.31)	0.67 (0.49–0.92)*	0.99 (0.36–2.69)
Rural	3465 (92.28)	290 (7.72)	1	1
Wealth index (n = 4677)				
*First tertile*	1599 (94.28)	97 (5.72)	1	1
*Second tertile*	1477 (90.28)	159 (9.72)	1.77 (1.36–2.31)*	1.54 (0.84–2.84)
*Third tertile*	1262 (93.83)	83 (6.17)	1.08 (0.80–1.47)	0.94 (0.36–2.45)
Family member owned bank account (n = 7063)				
*No*	3319 (94.69)	186 (5.31)	1	1
*Yes*	3330 (93.99)	213 (6.01)	1.21 (0.97–1.52)	1.05 (0.64–1.73)
Presence of waste pile near the home (n = 4668)				
*No*	3898 (93.01)	293 (6.99)	1	1
*Yes*	433 (90.78)	44 (9.22)	1.35 (0.97–1.88)	0.76 (0.39–1.48)
Main source of income (n = 3318)				
*Non-farming*	645 (94.57)	37 (5.43)	1	1
*Farming*	2429 (92.15)	207 (7.85)	1.48 (1.04–2.13)*	0.89 (0.31–2.54)
Ownership of the house (n = 4668)				
*Not owned*	631 (93.34)	45 (6.66)	1	1
*Owned*	3700 (92.69)	292 (7.31)	1.12 (0.80–1.53)	1.67 (0.62–4.44)
Family member owned mobile phone (n = 4657)				
*No*	1044 (94.74)	58 (5.26)	1	1
*Yes*	3276 (92.15)	279 (7.85)	1.53 (1.14–2.05)*	1.22 (0.64–2.31)
Source of drinking water (n = 4668)				
*Pipe within premises*	695 (95.73)	31 (4.27)	2.11 (1.44–3.09)*	0.67 (0.03–12.85)
*Improved*	2819 (91.41)	265 (8.59)	0.98 (0.56–1.72)	0.42 (0.02–9.41)
*Unimproved*	502 (95.8)	22 (4.20)	1.35 (0.75–2.43)	0.80 (0.04–15.38)
*Surface water*	315 (94.31)	19 (5.69)		
Location of drinking water source (n = 4668)				
*Own dwelling*	23 (88.46)	3 (11.54)	1	1
*Own compound*	731 (95.93)	31 (4.07)	0.32 (0.09–1.14)	0.05 (0.01–0.88)*
*Outside the compound*	3577 (92.19)	303 (7.81)	0.65 (0.19–2.17)	0.22 (0.03–1.42)
Boil drinking water (n = 1599)				
*No*	1397 (90.89)	140 (9.11)	1	1
*Yes*	59 (95.16)	3 (8.84)	0.51 (0.16–1.64)	1.41 (0.39–1.48)
Use chlorine (n = 1599)				
*No*	506 (90.84)	51 (9.16)	1	1
*Yes*	950 (91.17)	92 (8.83)	0.96 (0.67–1.37)	0.50 (0.28–0.89)
Use iodine (n = 1599)				
*No*	1370 (90.79)	139 (9.21)	1	1
*Yes*	86 (95.56)	4 (4.44)	0.46 (0.16–1.27)	0.25 (0.03–1.96)
Filter drinking water (n = 1599)				
*No*	1107 (90.59)	115 (9.41)	1	1
*Yes*	349 (92.57)	28 (7.43)	0.77 (0.50–1.19)	0.47 (0.21–1.04)
Type of toilet facility (n = 4668)				
*Improved*	1582 (92.68)	125 (7.32)	1	1
*Unimproved*	1614 (93.29)	116 (6.71)	0.91 (0.70–1.18)	1.24 (0.71–2.18)
*Open*	1135 (92.20)	96 (7.80)	1.07 (0.81–1.41)	1.18 (0.63–2.22)
Access to soap (n = 4668)				
*No*	508 (94.07)	32 (5.93)	1	1
*Yes*	3823 (92.61)	305 (7.93)	1.27 (0.87–1.84)	8.49 (1.15–62.73)*
Refuse disposal (n = 4668)				
*Unsafe*	2814 (92.84)	217 (7.16)	1	1
*Safe*	1517 (92.67)	120 (7.33)	1.03 (0.81–1.29)	1.13 (0.69–1.85)
*Distance from the nearest health facility*			1.00 (0.99–1.00)	1.00 (0.99–1.05)
*Number of household members*			1.04 (0.98–1.11)	1.03 (0.90–1.17)

aOR – adjusted odds ratio, CI – confidence interval, cOR – crude odds ratio

*P < 0.05.

Although children need ORS and more food and fluid during sickness, only a small proportion (6.90–20.00%) of children were given ORS, extra drinks (17.14–46.05%) and food (17.14–36.84%) (**Table 5**).

### Factors associated with treatment-seeking practices for diarrhoea

The bivariable analysis found that only the wealth index was associated with treatment-seeking practice for diarrhoea, while the multivariable model showed such a significant association for the sex of the child and wealth index. Female children had 63% (aOR = 0.37; 95% CI = 0.17–0.80) lower odds of being taken to health facilities for treatment of diarrhoea compared to male children. Children from households of second (aOR = 3.54; 95% CI = 1.37–9.18) and third (aOR = 8.60; 95% CI = 1.73–42.74) wealth tertiles had higher odds of being taken to a health facility for treatment of diarrhoea compared to children from households with the first (poorest) wealth tertile (**Table 7**).

**Table 7:** Factors associated with diarrhoea treatment-seeking practice for children <2 years of age, Birhan field site, 2018–19

	Treated for diarrhoea			
**Variable**	**No, n (%)**	**Yes, n (%)**	**cOR (95% CI)**	**aOR (95% CI)**
Sex				
*Male*	32 (16.49)	162 (83.51)	1	1
*Female*	32 (22.07)	113 (77.93)	0.70 (0.40–1.20)	0.37 (0.17–0.80)*
Residence				
*Urban*	7 (14.29)	42 (85.71)	1.47 (0.63–3.43)	1.10 (0.28–4.28)
*Rural*	57 (19.66)	233 (80.34)	1	1
Wealth index				
*First tertile*	31 (31.96)	66 (68.04)	1	1
*Second tertile*	27 (16.98)	132 (83.02)	2.3 (1.27–4.16)*	3.54 (1.37–9.18)*
*Third tertile*	6 (7.23)	77 (92.77)	6.03 (2.37–15.34)*	8.60 (1.73–42.74)*
Family member has bank account				
*No*	36 (23.08)	120 (76.92)	1	1
*Yes*	28 (15.47)	153 (84.53)	1.64 (0.95–2.84)	0.89 (0.40–2.00)
Ownership of the house				
*Not own*	6 (13.33)	39 (86.67)	1	1
*Own*	58 (19.68)	234 (80.14)	0.62 (0.25–1.53)	2.02 (0.41–9.87)
Family member owns mobile phone				
*No*	12 (20.69)	46 (79.31)	1	1
*Yes*	52 (18.64)	227 (81.36)	0.1.14 (0.56–2.3)	0.59 (0.21–1.68)
Main source of income				
*Non–farming*	4 (10.81)	33 (89.19)	1	1
*Farming*	40 (18.03)	167 (81.97)	0.51 (0.17–1.51)	0.98 (0.18–5.34)
Blood in diarrhoea				
*No*	60 (20.34)	235 (79.66)	1	1
*Yes*	4 (9.09)	40 (90.91)	0.39 (0.13–1.13)	0.57 (0.17–1.89)
*Number of household members*			0.94 (0.79–1.11)	1.06 (0.84–1.34)

aOR – adjusted odds ratio, CI – confidence interval, cOR – crude odds ratio

*P < 0.05.

## DISCUSSION

In total, 7.25% of the children in all the four rounds of data collection had history of diarrhoea. This finding was lower than a pooled estimate of diarrhoea prevalence of 12 sub-Saharan African countries [16], a 2016 Ethiopian DHS-based data analysis [17], and other studies in Bahir Dar City [16], Horo Guduru Wollega Zone [18], Kersa District, Eastern Ethiopia [19], and Dale District, Sidama Region [20]. One possible reason for this is that we exclusively included children <2 years of age, while the other studies included children aged 6–59 months. Diarrhoea prevalence was previously found to be higher among older children [17,21]. While continuous monitoring of diarrhoeal episodes would be ideal to accurately estimate the prevalence of diarrhoea, it was not feasible or realistic in our study due to financial constraints. Here we measured recall of diarrhoea in two weeks prior to each of the four study visits, a total of 8 weeks out of the 52-week period; however, it is likely that children had diarrhoea at other time points.

Although the difference was not significant, the proportion of children with diarrhoea was highest in the first round (11 September 2018 to 9 December 2018) and lowest in the second round (10 December 2018 to 9 March 2019). This might reflect seasonal variations, as September is the end of the rainy season which might have increased diarrhoea incidence. A longitudinal study in Eastern Ethiopia has also indicated that diarrhoea incidence is associated with seasonal variation [19].

We identified multiple factors associated with diarrhoea. Treating drinking water with chlorine was associated with less diarrhoea among children <2 years of age, meaning that children from households that treated drinking water with chlorine had lower odds of diarrhoea compared to children from households that did not. This finding is in line with other studies conducted in Ethiopia, which found water chlorination at the household level considerably reduced episodes of diarrhoea among children <5 years of age [22,23]. Household-based chlorination is the most cost-effective strategy as it is effective in killing many types of water-borne pathogens including parasites, fungi, bacteria and viruses [24].

Most children <2 years who had a history of diarrhoea were taken to health facilities for treatment. This finding was slightly higher than a study conducted from March 2015 to June 2016 in Mekelle City [25] and a systematic review from Ethiopia published in July 2018 [26]. The underlying reason could be the year when our study was conducted. Furthermore, there were no statistically significant differences in the proportion of children taken to health facility for treatment of diarrhoea by the time the data was collected, suggesting that health care seeking practice is not affected by seasonal variation.

ORS is the cornerstone of diarrhoea treatment in children. In our analysis, the proportion of children with diarrhoea who were given ORS ranged from 6% to 18%, which is lower than found in an analysis based on the 2016 Ethiopian DHS [27]. A systematic review in low and middle-income countries noted knowledge as a barrier for ORS uptake among children with diarrhoea [28]. This could have been because of a lower level of awareness about ORS therapy among community members and caretakers. Moreover, a previous study conducted in Ethiopia showed that nearly 50% mothers restricted fluids when their children developed diarrhoea [28]. In our research, only 40% children with diarrhoea were given more food and liquids intake. This finding was lower than that of other studies conducted in Ethiopia [29,30], possibly due to the difference in the study population (i.e. we study included only children <2 years of age, while the other studies included all children <5 years) and setting (urban vs rural) [31]. Moreover, prior research found that a lack of knowledge about diarrhoea to be the main reason for mothers restricting food and liquids for children with diarrhoea [31–33].

There were multiple factors associated with care seeking, including sex and wealth. We observed that female children had lower odds of being taken to health facility for diarrhoea treatment compared to their male counterparts, which follows the findings of a study in Ethiopia where female children had higher odds of delayed treatment for diarrhoea compared to male children [34]. Gender inequality could be the possible reason for this observed, gender-based difference [24]. Furthermore, children from wealthy families had higher odds of being taken to health facilities for diarrhoea treatment compared to their counterparts from the poorest family. This finding is similar to that of the aforementioned study conducted in Ethiopia which indicated that children from households with the lowest wealth quintile had higher odds of delayed treatment for diarrhoea [34]. The reason for this may be lack of assets among parents to cover the expense of user fees/medicine, travel costs to and from health facilities [34].

Notably, most findings on the proportion of children with diarrhoea and their treatment-seeking practices from Ethiopia come from cross-sectional studies that provide only a snapshot in time. However, as treatment-seeking practices and prevalence of diarrhoea vary from time to time, such studies fail to show this variation and may provide either overestimates or underestimates of the true values. Most of these studies included all children <5 years of age.

This analysis was based on a large sample size acquired through repeated cross-sectional surveys. which enabled us to explore the trends of diarrhoea prevalence and treatment-seeking practice for diarrhoea. However, recall bias might have affected our findings, since the data collection depended on the mothers’ report of diarrhoea and treatment-seeking practices in the two weeks before each round of surveys. However, we mitigated this possibility by limiting the recall period to two weeks prior to the survey visit. Moreover, mothers also may have failed to report mild diarrhoea cases.

## CONCLUSIONS

In our analysis, treatment-seeking practices for children with diarrhoea were higher compared to previous studies conducted in Ethiopia, although a substantial proportion of children with diarrhoea were still not treated, while a significant proportion of children with diarrhoea who sought care had not received the recommended treatments such as ORS and extra fluid during diarrhoea episodes. Female children and children from the poorest families had lower odds of being taken to health facilities for diarrhoea treatment. This highlights the importance of raising awareness to improve knowledge of caretakers about diarrhoea management. Further studies on this topic should include other variables that we had not collected in our research, while further qualitative explorations could identify if there is sex preference in treatment seeking for children with diarrhoea.

## Additional material


Online Supplementary Document
