# Supplementary material for: Prevalence of diarrhoea and treatment-seeking practices among children <2 years of age in the Birhan cohort, Ethiopia, 2018–19
Source: J Glob Health. 2024 Nov 1;14:04181. doi: 10.7189/jogh.14.04181 (PMC11531709; doi:10.7189/jogh.14.04181)
Supplement: Online Supplementary Document [file jogh-14-04181-s001.pdf]

Table S1. Household, sociodemographic, housing and water, sanitation and hygiene characteristics of enrolled children < 2 Birhan HDSS, Ethiopia, 2018-2019

**Variables**	**Number**	**Percent**
Residence (N=4,677)		
• Urban	922	19.71
• Rural	3755	80.29
**Agro-ecology** (N=4,677)		
• Highland	1791	38.29
• Lowland	2889	61.71
Ethnicity (N=3,338)		
• Amhara	3027	90.68
• Oromo	202	6.05
• Other	109	3.27
Wealth index (N=4,677)		
• First tertile (least wealthy)	1696	36.26
• Second tertile	1636	34.98
• Third tertile (wealthiest)	1345	28.76
Household member with bank account (N=4657)		
• No	2358	50.63
• Yes	2285	49.07
• Unknown	14	0.30
Family has own income (N= 4657)		
• No	110	2.36
• Yes	4547	97.64
Main source of income or livelihood (N= 3,318)		
• Government employment	141	4.25
• Private employment	52	1.57
• NGO employment	30	0.90
• Farming	2636	79.45
	62	1.87

• Petty trader<br>• Merchant<br>• Daily labor<br>• Other	105<br>218<br>74	3.16<br>6.57<br>2.23
Ownership of the house (N=4,668)<br>• Own<br>• Rented from government<br>• Rented from individual<br>• Other	 3992<br>50<br>590<br>36	 85.58<br>1.07<br>12.64<br>0.77
Waste pile near the home (**N=**4668)<br>• No<br>• Yes	 4191<br>477	 89.78<br>10.22
Family member owned mobile phone (N= 4657)<br>• No<br>• Yes	 1102<br>3555	 23.66<br>76.34
Source of drinking water (N=4668)<br>• Within premises<br>• Improved<br>• Unimproved<br>• Surface water	 726<br>3084<br>524<br>334	 15.55<br>66.07<br>11.23<br>7.16
Location of drinking water source (N= 4668)<br>• Own dwelling<br>• Own compound<br>• Outside the compound	 26<br>762<br>3880	 0.56<br>16.32<br>83.12
Drinking water treated (N= 4668)<br>• Yes<br>• No	 1599<br>3069	 34.25<br>65.75

Drinking water treated by boiling (N= 1599)<br>• No<br>• Yes	 1531<br>62	 96.12<br>3.88
Drinking water treated by chlorine (N= 1599)<br>• No<br>• Yes	 557<br>1042	 34.83<br>65.17
Drinking water treated by iodine (N= 1599)<br>• No<br>• Yes	 1509<br>90	 94.37<br>5.63
Drinking water treated by filtration (N= 1599)<br>• No<br>• Yes	 1222<br>377	 76.42<br>23.58
Water treatment options (N= 1599)<br>• None of the above<br>• At least one of the above	 185<br>1414	 11.57<br>88.43
Type of toilet facility (N= 4668)<br>• Improved<br>• Unimproved<br>• Open	 1707<br>1730<br>1231	 36.57<br>37.06<br>26.37
Access to soap (4668)<br>• No<br>• Yes	 540<br>4128	 11.57<br>88.43
Refuse disposal (N= 4668)<br>• Pit<br>• Open field<br>• Burn<br>• Compost	 879<br>1170<br>1226<br>672<br>86	 18.83<br>25.06<br>26.26<br>14.40<br>1.84

• Municipality collection<br>• Farm<br>• Other	619<br>16	13.26<br>0.34
Estimated walking time (in minutes) to the nearest health facility (N= 4668)	Mean ± SD	65.74±54.05
Family size (3941)	Mean + SD	4.79± 1.85
Vaccinated at least once (N=4190)<br>• No<br>• Yes	 139<br>4051	 3.32<br>96.68
Vaccinated with rota1 (N= 2020)<br>• No<br>• Yes	 85<br>1935	 4.21<br>95.79
Vaccinated with rota2 (N= 2009)<br>• No<br>• Yes	 165<br>1844	 8.21<br>91.79

Table S2. Housing, child, care giver, water, sanitation and hygiene characteristics of children < 2 with diarrhea, Birhan HDSS, 2018-2019

**Variables**	**Number (%)**
Ethnicity (n=246)	
• Amhara	231(93.90)
• Oromo	10(4.07)
• Other	5(2.03)
Wealth index (N=339)	
• First tertile (least wealthy)	97(28.61)
• Second tertile	159(46.90)
• Third tertile (wealthiest)	83(24.48)
One member of the household owned bank account (N=337)	
• No	156(46.29)
• Yes	181(53.71)
Family has own income (N=337)	
• No	7(2.08)
• Yes	330(97.92)
Main source of income or livelihood (N= 244)	
• Non-farming	37(15.16)
• Farming	207(84.84)
Ownership of the house (N=337)	
• Not owned	45(13.35)
• Owned	292(86.65)
Waste pile near the home (N=337)	
• No	293(86.94)
• Yes	44(13.06)
Owning mobile phone by member of the household (N=337)	
• No	58(17.21)

• Yes	279(82.79)
Source of drinking water (N=337)<br>• Pipe within premises<br>• Improved<br>• Unimproved<br>• Surface water	 31(9.20)<br>265(78.84)<br>22(6.53)<br>19(5.64)
Location of drinking water source (N=337)<br>• Own dwelling<br>• Outside the compound	 34(10.09)<br>303(89.91)
Treat drinking water (N=337)<br>• Yes<br>• No	 194(57.57)<br>143(42.43)
Treat drinking water by boiling (N=143)<br>• No<br>• Yes	 140(97.90)<br>3(2.10)
Use chlorine to treat drinking water (N= 143)<br>• No<br>• Yes	 51(35.66)<br>92(64.34)
Use iodine to treat drinking water (N= 143)<br>• No<br>• Yes	 139(97.20)<br>4(2.80)
Filter to treat your drinking water (N=143)<br>• No<br>• Yes	 115(80.42)<br>28(19.58)
Used at least one water treatment options used (N=143)<br>• No<br>• Yes	 26(18.18)<br>117 (81.82)

Type of toilet facility (N=337)<br>• Improved<br>• Unimproved<br>• Open	 125(37.09)<br>116(34.42)<br>96(28.49)
Access to soap (N=337)<br>• No<br>• Yes	 32(9.50)<br>305(90.50)
Refuse disposal (N=337)<br>• Unsafe<br>• Safe	 217(64.39)<br>120(35.61)
Vaccinated at least once (N=326)<br>• No<br>• Yes	 7(2.15)<br>319(97.85)
Vaccinated rota1 (N=155)<br>• No<br>• Yes	 4(2.58)<br>151(97.42)
Vaccinated rota2 (N=152)<br>• No<br>• Yes	 6(5.95)<br>146 (96.05)
